# Transumbilical single-site laparoscopic parallel mattress suturing prevents bleeding and chronic pelvic pain in myomectomy: a retrospective cohort study of 124 cases with intramural fibroids

**DOI:** 10.1186/s12893-022-01626-8

**Published:** 2022-05-13

**Authors:** Xi Zeng, Lin Li, Hui Ye, Mingrong Xi

**Affiliations:** 1 Gynecology Department, West China Second University Hospital, Sichuan University, Chengdu, 610041 China; 2 Key Laboratory of Birth Defects and Related Diseases of Women and Children (Sichuan University), Ministry of Education, Sichuan, 610041 China

**Keywords:** Myomectomy, Parallel mattress suture, Adhesion, Bleeding, Single-site laparoscopy

## Abstract

**Background:**

The most common complications of myomectomy are intraoperative hemorrhage and postoperative adhesion. The key point to overcome this problem is to improve suture quality. However, to date, there is still no consensus on the optimal method of uterine repair. In this study, we explored the effectiveness and feasibility of single-site laparoscopic parallel mattress sutures to reduce intraoperative bleeding and postoperative adhesion.

**Methods:**

A retrospective cohort analysis was performed, according to the STROBE guidelines, on 124 patients with intramural fibroids admitted between May 2020 and April 2021. The cases were divided into two groups based on the description of the uterine incision suture in the surgical records, including 68 cases in the parallel mattress suture (PMS) group and 56 cases in the simple continuous suture (SCS) group. Operation-related indicators, bleeding indicators, surgical complications, scar reduction index 1 month after surgery, and the incidence of chronic pelvic pain 6 months after the surgery were observed. Independent sample t-tests and Mann–Whitney U tests were performed for the measurement data, and Pearson Chi-square tests were performed for count data. Statistical significance was set at P < 0.05.

**Results:**

There was no significant difference in the baseline characteristics between the two groups. All operations were performed under transumbilical single-site laparoscopy without conversion. Compared to the SCS group, the PMS group had earlier postoperative anal exhaust (14.3 ± 6.7 h vs. 19.2 ± 9.6 h, P = 0.002), fewer postoperative hemoglobin drops (7.6 ± 3.7 g/L vs. 11.6 ± 4.3 g/L, P = 0.000), smaller uterine scars (3.7 ± 1.9 cm vs. 5.2 ± 1.8 cm, P = 0.000), and a larger uterine scar reduction index (50.2% vs. 31.0%, P = 0.000) one month after surgery and less chronic pelvic pain 6 months after surgery (2.9% vs. 12.5%, P = 0.016). No difference was found in auxiliary trocar usage, transfusion rate, operation time, hospital stay, or perioperative complications between the two groups.

**Conclusion:**

Seromuscular parallel mattress sutures during myomectomy can prevent pinhole errhysis of the uterine incision, achieve complete serosal and aesthetic incisions, and reduce postoperative chronic pelvic pain. It is effective and feasible to complete a parallel mattress suture during myomectomy via single-site laparoscopy. Further prospective studies are required to determine its efficacy as well as pregnancy outcomes.

## Background

Uterine leiomyomas, also commonly known as fibroids, are the most common gynecologic benign pelvic tumors, with an incidence of 60% in reproductive-aged women and 70% in women over 30 years of age [1, 2]. Approximately 30% of them will present with severe symptoms, which can include abnormal uterine bleeding, anemia, pelvic pain and pressure, back pain, urinary frequency, constipation, or infertility, and will require intervention [1, 2]. Medical treatments include gonadotropin-releasing hormone agonists (GnRHa), gonadotropin-releasing hormone antagonists (GnRHant), selective progesterone receptor modulators (SPRMs), etc. In addition to drug side effects, the disappearance of efficacy after drug withdrawal limits its clinical application, although a few drugs, such as elagolix and ulipristal, have shown encouraging results in clinical trials [1]. Myomectomy is most often offered to patients who desire future fertility but is also considered by those who have completed childbearing and wish to retain their uterus [2].

The most common complications of myomectomy are hemorrhage and adhesion, among which the most inconvenient hemorrhage is incision pinhole errhysis [3]. The mechanisms of adhesion include exposure of sutures, evagination of the fibroid pseudocapsule, and bleeding from the incision [4, 5]. Adhesions, which form in 60–80% of all abdominal operations, in 60–90% of gynecological surgeries, and in 23–88% of myomectomy, can be associated with severe complications such as small bowel obstruction, chronic pelvic pain, complications in further operations, or impaired fertility [5–7]. Myomectomy has also been associated with uterine rupture and obstetrical outcomes [8].

The key point to overcome the abovementioned problem is to improve suture quality [4, 6, 9]. However, to date, there is still no consensus on the optimal method of uterine repair. Shah et al. [10] deemed uterine closure by single-layer modified mattress sutures during cesarean section to be superior in preventing uterine scar dehiscence in future pregnancy compared to conventional single-layer running sutures. The protruding wounds might be in contact with other organs for a longer period, which might be a reason for an increasing degree of adhesion development [11]. Pellicano et al. [12] considered that in myomectomy, the rate of adhesions was significantly higher in patients treated with interrupted “8” sutures than with subserous sutures.

In myomectomy, laparoscopy has several well‑known advantages over laparotomy, such as less pain, less blood loss, and shorter hospital stay [13]. Single-site laparoscopic myomectomy is comparable to conventional laparoscopic myomectomy in terms of safety and feasibility and more advantageous in terms of immediate postoperative pain [14–16].

In this study, we studied the effects of different uterine incision suture methods, parallel mattress suture (PMS) vs. simple continuous suture (SCS), during myomectomy on perioperative indicators, bleeding indicators, postoperative uterine incision recovery, and postoperative chronic pelvic pain, with the aim of improving uterine incision suture techniques and reducing surgical bleeding and postoperative adhesion. To the best of our knowledge, this study is the first study in the world on parallel mattress suture for myomectomy incision through a single-site laparoscopy.

## Methods

### General information

This study was conducted according to the STROBE guidelines, and approved by the Institutional Review Board and Ethics Committee (Number 2021-187) of West China Second University Hospital, Sichuan University. All patients gave consent before project implementation. Patients were informed of all treatment options before surgery and that if single-port laparoscopy was difficult, auxiliary pores may be added or even converted to laparotomy when necessary.

The clinical data of 124 cases of intramural uterine fibroids (International Federation of Gynecology and Obstetrics (FIGO) classification type III–VI) [17] admitted to the teaching hospital from May 2020 to April 2021 were retrospectively analyzed. The cases were divided into two groups based on the description of the uterine incision suture in the surgical records, including 68 cases in the parallel mattress suture (PMS) group and 56 cases in the simple continuous suture (SCS) group.

### Inclusion and exclusion criteria

#### Inclusion criteria

The ultrasound diagnosis was intramural fibroid of the uterus (III–VI), the fibroid diameter was 5–10 cm, and the number of fibroids was less than five.

#### Exclusion criteria

Type 0–II, VII, and VIII uterine fibroids, patients complicated with liver, kidney, heart, and lung dysfunction, patients with coagulation dysfunction, malignant tumors, mental illness, lactation or pregnancy, and patients receiving hormone therapy before surgery were excluded.

### Surgical techniques

**Fig. 1:**
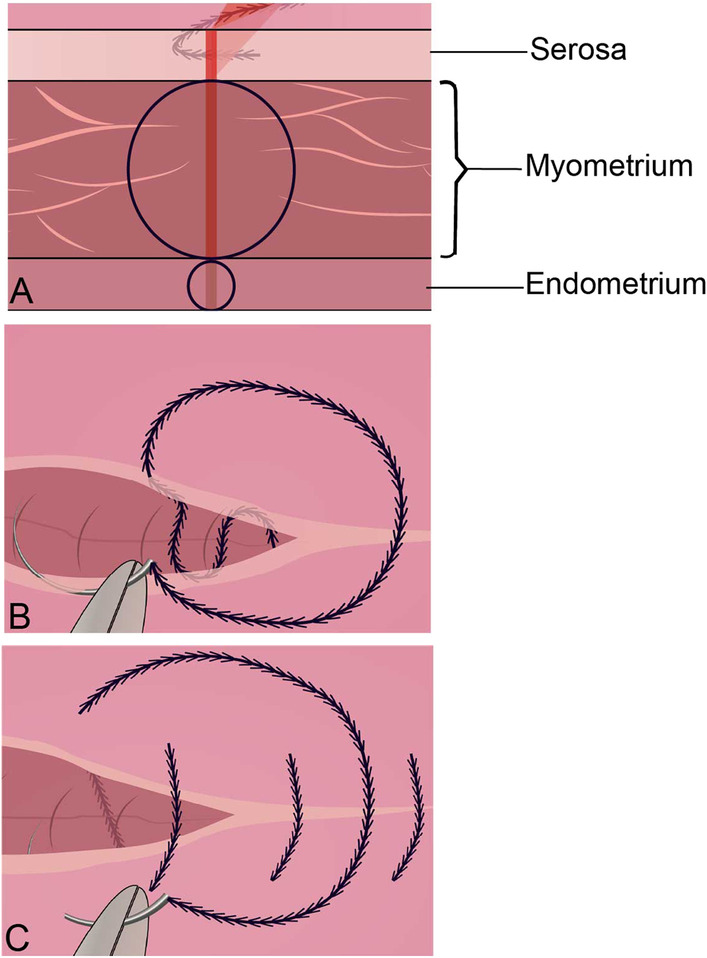
Schematic diagram of suturing uterine defect. **A** Suture layer by layer on uterine defect. **B** Continuous parallel mattress suture (PMS) on the seromuscular layer. **C** Simple continuous suture (SCS) on the seromuscular layer

All operations were performed by the same three surgeons, Xi Zeng, Lin Li and Hui Ye. From November 2020 onward, all myomectomies were performed with PMS. The patient was placed in the Trendelenburg position and sterilized after general anesthesia and tracheal intubation. A 2-cm incision was made along the umbilicus, a port channel was placed, and an artificial pneumoperitoneum with a pressure of 14 mmHg was established. If the operation was difficult to perform, a 5 mm trocar was added to the left lower abdomen. If posterior wall fibroids were encountered, a simple uterine-lifting apparatus or suspension uterus via the abdominal wall was used to expose the fibroids. Six units of pituitrin dissolved in 20 mL normal saline (1:20) were injected into the myometrium 1 cm next to the fibroid base using a long puncture needle. The tumor pseudocapsule was incised with a monopole hook, the fibroid was clamped with grasping forceps, the pedicle was coagulated and cut off using bipolar forceps, and the fibroid was removed. According to the depth of the fibroid location, as shown in Fig. 1A, the uterine incision was sutured in 2–3 layers. The endometrium was continuously sutured with a 3–0 absorbable line if the uterine cavity was penetrated during the removal of fibroids. The myometrium was closed by continuous suturing with a 2–0 absorbable barbed line. The seromuscular layer was sutured with a 2–0 absorbable barbed line by continuous parallel mattress suture (PMS, Fig. 1B) or simple continuous suture (SCS, Fig. 1C).

#### Parallel mattress suture (PMS)

As shown in Fig. 1B, continuous parallel mattress suture was performed on the seromuscular layer with 2–0 absorbable barbed sutures. The first needle was inserted from the top of the uterine incision, parallel to the incision, and alternately passed through the seromuscular layer below the edge of the incision on both sides, and the suture was gradually tightened to the end of the incision. If the incision tension was large, the seromuscular layer could be sutured again using the same method until the incision was completely edge-to-edge closed.

#### Simple continuous suture (SCS)

As shown in Fig. 1C, simple continuous suture was performed on the seromuscular layer with a 2–0 absorbable barbed line. The first needle was inserted into the serosal surface 5 mm from the top of the uterine incision, and the needle was removed from the serosal surface 5 mm from the opposite side of the incision. The needle gap was 10 mm. After the stitches were successively sutured 5 mm to the end of the incision, they were cut along the uterine surface. If there was pinhole errhysis, bipolar electrocoagulation could be performed, or a single or “8” shape suture was conducted with a 3–0 absorbed line. If subtle bleeding still existed, an absorbable hemostatic gauze was taken to cover the pinhole, and a drainage tube was placed on the pelvic floor through the posterior vaginal fornix.

After suturing the uterine defect, the fibroid was placed in a homemade specimen bag and removed using a cold knife through the umbilical port to avoid tumor dissemination. Finally, the umbilical incision was reshaped with 2–0 absorbable suture. After the surgery, the patients were treated with an intravenous oxytocin drip for 24–48 h. If the fibroids had penetrated the uterine cavity, cephalosporin was administered for 24–48 h to prevent infection.

### Observation indicators

Information on all patients was recorded, including operation time, postoperative exhaust time, postoperative hospital stay, estimated surgical blood loss, hemoglobin decline (ΔHb) (complete blood cell count was performed in all patients within 1 week before surgery and 1 days after the surgery, and the hemoglobin decline was calculated), transfusion rate, postoperative complications including surgical site infection (SSI), fever (T ≥ 38 °C), and intestinal obstruction.

### Follow-up

During outpatient follow-up one month after surgery, ultrasound (GE Voluson E8, probe type of EUP-V53 W, frequency of 5–7.5 MHz) was used to detect uterine scar length, and the scar reduction index (SRI) was calculated using the formula: SRI = (preoperative maximal fibroid diameter − scar length)/preoperative maximal fibroid diameter × 100%.

Six months after the operation, each patient was followed-up by means of outpatient visits or telephone calls to inquire whether chronic pelvic pain had occurred.

### Statistical analysis

The data were analyzed using IBM SPSS Statistics 22.0 (Chicago, IL, USA). The measurement data are expressed as the mean ± standard deviation (*x* ± *s*). First, the one-sample Kolmogorov–Smirnov test and Levene’s test were carried out to determine whether the data conformed to a normal distribution and homogeneity of variance, and the test level was set at 0.1. Finally, body mass index (BMI), fibroid size, and hospital stays corresponded to a normal distribution (P > 0.1), and an independent sample t test was carried out, whereas other variables were in an abnormal distribution, then, the Mann–Whitney U test was performed. The counting data were expressed as n (%), and Pearson’s chi-square test was performed. The missing values were interpolated by regression estimation. A two-tailed P value was used, and the difference was considered statistically significant if P < 0.05.

## Results

### The general characteristics

The age of the PMS group was 35.7 ± 8.5 years old, and the diameter of fibroids was 6.4 ± 1.5 cm. The age of the SCS group was 36.8 ± 9.1 years old, and the fibroid diameter was 6.9 ± 1.6 cm. As shown in Table 1, there was no significant differences in the baseline characteristics between the two groups. Four of 68 (5.9%) patients in the PMS group were lost to follow-up, and 2/56 (3.6%) patients in the SCS group were lost to follow-up.

**Table 1:** Baseline characteristics between the two groups (*x* ± *s* or n (%))

		PMS^a^ (n_1_ = 68)	SCS^b^ (n_2_ = 56)	P value
Age (year)		35.7 ± 8.5	36.8 ± 9.1	0.514
BMI (kg/m^2^)		21.2 ± 3.4	20.7 ± 2.8	0.873
Fibroid size (cm)		6.4 ± 1.5	6.9 ± 1.6	0.659
Fibroid type^c^	III	19 (27.9%)	18 (32.1%)	0.392
	IV	12 (17.6%)	13 (23.2%)	
	V	17 (25.0%)	11 (19.6%)	
	VI	20 (29.4%)	14 (25.0%)	
Fibroid site	Anterior	18 (26.5%)	6 (10.7%)	0.187
	Posterior	20 (29.4%)	19 (33.9%)	
	Fundus	16 (23.5%)	21 (37.5%)	
	Lateral	14 (20.6%)	10 (17.9%)	
Preoperative Hb (g/L)		105.3 ± 17.2	108.5 ± 19.9	0.353
Lost of follow-up^d^		4 (5.9%)	2 (3.6%)	0.458

^a^PMS: parallel mattress suture

^b^SCS: simple continuous suture

^c^According to FIGO Leiomyoma Subclassification System [17]

^d^Chronic pelvic pain was followed-up 6 months after the operation

### Visual effect of surgery

**Fig. 2:**
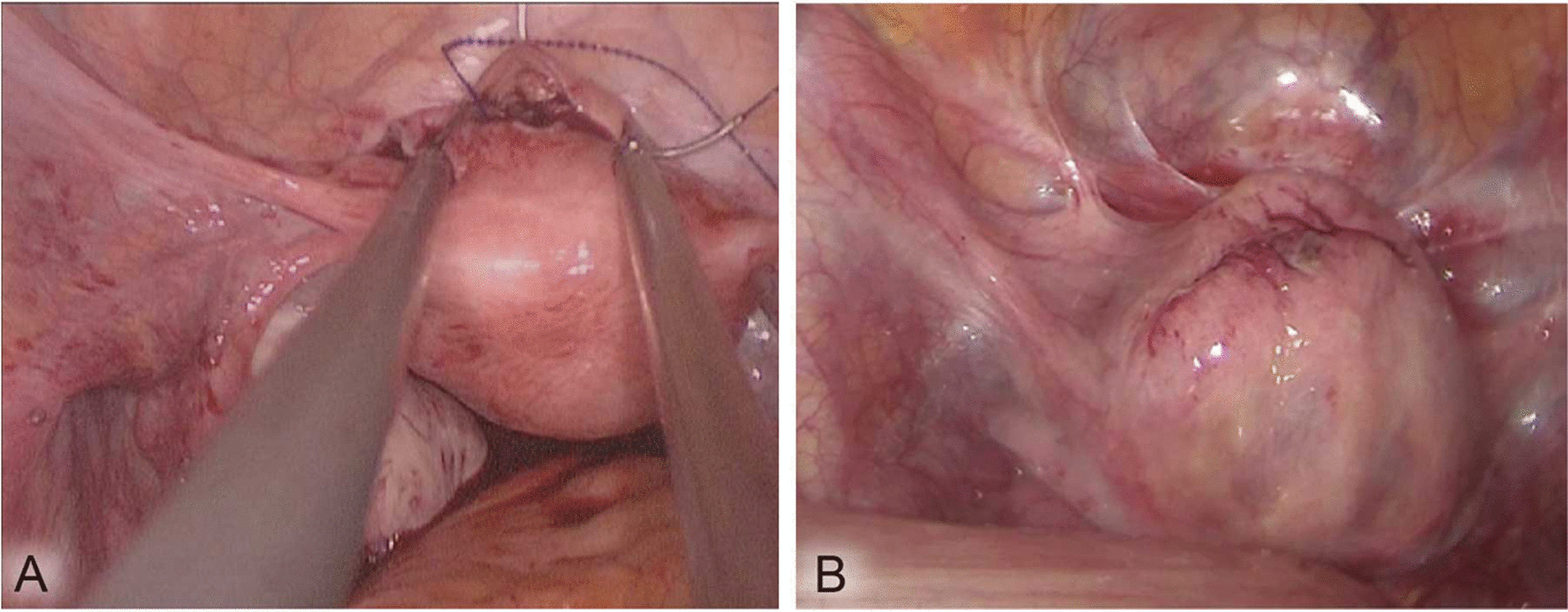
Surgical images of the PMS group. **A** Continuous parallel mattress suturing of the uterine seromuscular layer. **B** Appearance of the uterus after suturing: no suture exposure or pinhole errhysis at the incision

Anatomical closure of the incision on the uterus was performed. As shown in Fig. 2, in the PMS group, after seromuscular continuous parallel mattress suturing (Fig. 2A), there were no sutures exposed or pinhole errhysis on the surface of the uterus (Fig. 2B).

### Perioperative indicators

All operations were performed under transumbilical single-site laparoscopy without conversion to laparotomy. As shown in Table 2, compared with the SCS group, the PMS group had earlier postoperative anal exhaust (14.3 ± 6.7 h vs. 19.2 ± 9.6 h, P = 0.002), less postoperative hemoglobin decline (7.6 ± 3.7 g/L vs. 11.6 ± 4.3 g/L, P = 0.000). However, the frequency of auxiliary trocar use (5.8% vs. 3.6%, P = 0.282), operation time (76.9 ± 27.2 min vs. 75.1 ± 24.8 min, P = 0.810), estimated blood loss (115.4 ± 86.6 mL vs. 194.0 ± 78.6 mL, P = 0.518), blood transfusion rate (7.4% vs. 10.7%, P = 0.516), and postoperative hospital stay (1.9 ± 0.8 day vs. 2.1 ± 0.8 day, P = 0.299) were not significantly different between the two groups.

**Table 2:** Perioperative indicators ($\overline{x }\pm$ s or n (%))

	PMS^a^ (n_1_ = 68)	SCS^b^ (n_2_ = 56)	P value
Auxiliary trocar	4 (5.8%)	2 (3.6%)	0.282
Operation time (min)	76.9 ± 27.2	75.1 ± 24.8	0.810
Initial anal exhaust (h)	14.3 ± 6.7	19.2 ± 9.6	0.002
Hospital stay (days)	1.9 ± 0.8	2.1 ± 0.8	0.299
ΔHb^c^ (g/L)	7.6 ± 3.7	11.6 ± 4.3	0.000
EBL^d^ (mL)	115.4 ± 86.6	194.0 ± 78.6	0.518
Transfusion rate	5 (7.4%)	6 (10.7%)	0.516

^a^PMS: parallel mattress suture

^b^SCS: simple continuous suture

^c^∆Hb refers to preoperative hemoglobin minus postoperative hemoglobin

^d^EBL: estimated blood loss

### Postoperative complications

According to the guidelines for surgical site infection (SSI) [18], the grade of all SSIs in this study was superficial SSI at umbilical single port incision. Six months after the operation, each patient was followed-up with outpatient visits or telephone calls to inquire whether chronic pelvic pain had occurred. As shown in Table 3, the incidence of chronic pelvic pain six months after surgery was lower in the PMS group than in the SCS group (2.9% vs. 12.5%, P = 0.016). There were no significant differences in SSI (1.8% vs. 5.9%, P = 0.252), postoperative fever (4.4% vs. 10.7%, P = 0.181), or intestinal obstruction (0% vs. 1.8%, P = 0.671).

**Table 3:** Postoperative complications (n (%))

	PMS^a^ (n_1_ = 68)	SCS^b^ (n_2_ = 56)	P value
SSI^c^	1 (1.8%)	4 (5.9%)	0.252
Postoperative fever^d^	3 (4.4%)	6 (10.7%)	0.181
Intestinal obstruction	0 (0%)	1 (1.8%)	0.671
Chronic pelvic pain	2 (2.9%)	7 (12.5%)	0.016

^a^PMS: parallel mattress suture

^b^SCS: simple continuous suture

^c^SSI: surgical site infection

^d^Ear temperature was more than 38 ℃

### Uterine incision healing evaluation by ultrasound

**Fig. 3:**
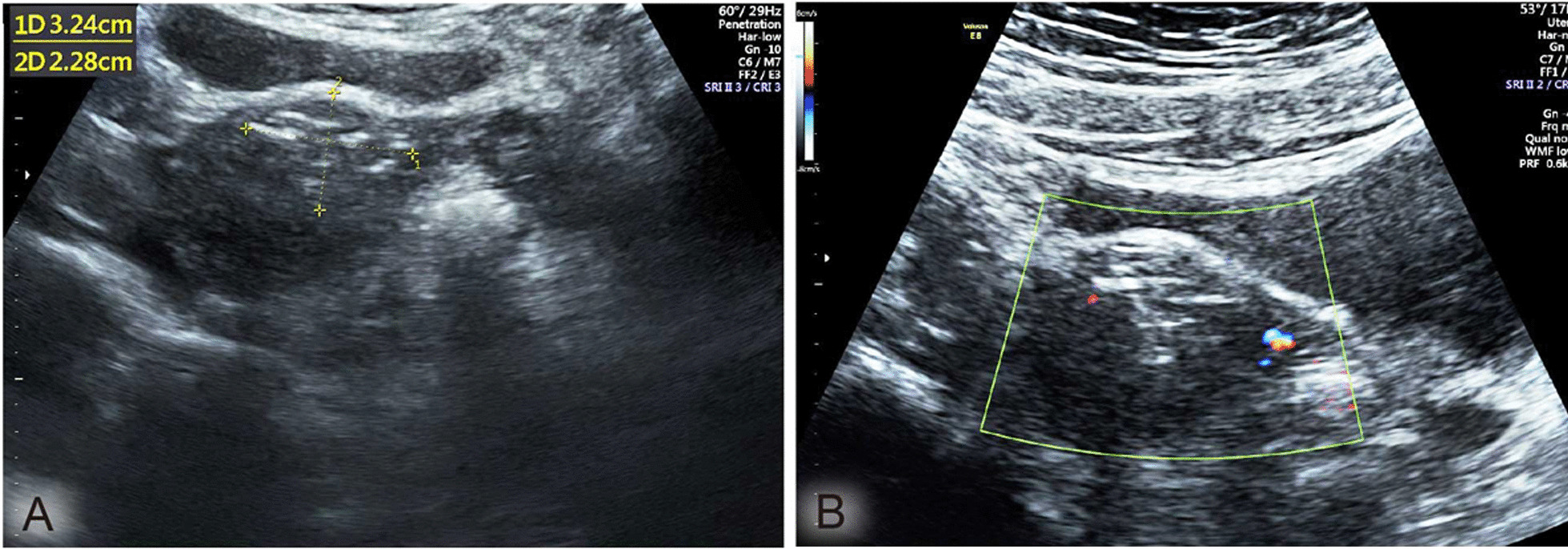
Sonography one month after the surgery: the myomectomy scar showed an ill-defined heterogeneous myometrial texture (**A**) with a slightly higher vascular signal compared with the surrounding myometrium (**B**). The maximum diameter line of the scar was measured to calculate the SRI

One month after surgery, ultrasound (GE Voluson E8, probe type of EUP-V53 W, frequency of 5–7.5 MHz) was used to detect uterine scar length. For evaluation, the myometrial scar diameter was compared with the maximal myometrial diameter of the area previously occupied by the uterine fibroid before surgery [19]. As shown in Fig. 3, the scar reduction index (SRI) was calculated using the formula SRI = (preoperative maximal fibroid diameter − scar length)/preoperative maximal fibroid diameter × 100%. As shown in Table 4, compared with the SCS group, the PMS group had a smaller uterine scar length (3.7 ± 1.9 cm vs. 5.2 ± 1.8 cm, P = 0.000) and larger SRI (50.2% vs. 31.0%, P = 0.000), as measured by ultrasound one month after surgery.

**Table 4:** Uterine incision healing evaluation by ultrasound ($\overline{x }\pm$ s or n (%))

	PMS^a^ (n_1_ = 68)	SCS^b^ (n_2_ = 56)	P value
Uterine scar length(cm)	3.7 ± 1.9	5.2 ± 1.8	0.000
SRI^c^ (%)	50.2 ± 15.4	31.0 ± 11.8	0.000

^a^PMS: parallel mattress suture

^b^SCS: simple continuous suture

^c^SRI: scar reduction index, SRI = (preoperative maximal fibroid diameter − scar length)/preoperative maximal fibroid diameter × 100%

## Discussion

The most common complications of myomectomy are hemorrhage and adhesion, among which the most inconvenient hemorrhage is incision pinhole errhysis [3]. The mechanism of adhesion includes exposure of sutures, evagination of the fibroid pseudocapsule, and bleeding from the incision [4, 5, 7]. The key to overcome the abovementioned problems is improving suture skills [9]. To solve the problem of postoperative adhesion caused by incision pinhole errhysis and incomplete serosal incision during myomectomy, we proposed a new suture method, that is, continuous parallel mattress suture of the seromuscular layer, as shown in Fig. 1B, which achieved satisfactory results with no incision pinhole errhysis, complete serosal incision, and prevention of postoperative adhesion. To the best of our knowledge, this study is the first study in the world on parallel mattress suture of myomectomy incision through a single-site laparoscopy.

In myomectomy, the problem of incision pinhole errhysis is often encountered after the closure of the uterine incision, and hemostasis can only be achieved by electrocoagulation, restitching, and using hemostatic materials. In this process, if attention is not paid during electrocoagulation, the previous suture may be broken by thermal damage or even need to be sutured again, which not only increases the operation time but also increases the possibility of new pinhole errhysis and sometimes even falls into a cycle of repeated pinhole errhysis-suturing-new pinhole errhysis-suturing. Even if the incision bleeding is stopped during the intraoperative examination, postoperative incision bleeding may still occur after the disappearance of carbon dioxide pneumoperitoneum and loosening of sutures due to uterine contraction, which can explain why postoperative hemoglobin drops were usually greater than the estimated blood loss. In this study, as shown in Fig. 2B, since there was no pinhole around the incision after continuous parallel mattress suturing of the seromuscular layer of the uterine incision, pinhole errhysis was not observed. The hemoglobin drop (7.6 ± 3.7 g/L) on the first day after surgery in the PMS group was significantly lower than that in the SCS group (11.6 ± 4.3 g/L), P = 0.000. Therefore, continuous parallel mattress suturing of the seromuscular layer can effectively solve the problem of incision pinhole errhysis. However, it is worth noting that the premise of satisfactory results of continuous parallel mattress suturing of the seromuscular layer must be that the muscular layer is firmly sutured to close the tumor cavity, and no obvious active bleeding exists. Otherwise, although no pinhole exists on the incision surface after parallel mattress suturing, incision bleeding will still occur just below the incision.

It is estimated that 60–90% of women will have adhesions after pelvic surgery [7]. Postoperative adhesions mainly manifested as chronic pelvic pain, secondary infertility, intestinal obstruction, and difficulty in reoperation. Adhesion formation mechanisms include suture exposure, fibroid pseudocapsule evagination, and incision bleeding [4, 20]. The key points to solve the above problems are to improve suture skills, reduce suture exposure, restore anatomy with meticulous sutures, and repair tissue defects [5, 9]. In light of the problems resulting in pelvic adhesion after myomectomy, we found that parallel mattress suturing of the seromuscular layer, as shown in Fig. 2B, can achieve no suture exposure, prevent pinhole errhysis, and achieve complete serosal incision. Ultrasound showed that the length of the uterine scar in the PMS group (3.7 ± 1.9 cm) was lower than that in the SCS group (5.2 ± 1.8 cm), P = 0.000, and the SRI in the PMS group (50.2%) was greater than that in the SCS group (31.0%), P = 0.000. Six months after the surgery, chronic pelvic pain was significantly lower in the PMS group (2.9%) than in the SCS group (12.5%) (P = 0.016). It is suggested that parallel mattress suturing of the uterine seromuscular layer can reduce uterine scarring and postoperative chronic pelvic pain and has a positive effect on the prevention of postoperative adhesions.

Enhanced recovery after surgery (ERAS) has been favored in almost all surgical fields. Gastrointestinal function recovery is one of the most important evaluation indices in ERAS, and postoperative anal exhaust is the first indicator of postoperative gastrointestinal function recovery. The factors affecting anal exhaust after operation include abdominopelvic exudation, operation time, and anesthesia. In this study, pelvic drainage was not generally performed because of the single-site laparoscopic approach. Although postoperative abodominopelvic exudation could not be measured, the patients in the PMS group had earlier anal exhaust than those in the SCS group (14.3 ± 6.7 h vs. 19.2 ± 9.6 h, P = 0.002), indirectly indicating that the postoperative uterine incision exudation was less in the PMS group than in the SCS group since there was no suture exposure or pinhole errhysis.

Uterine incision healing after myomectomy has a significant impact on the safety of pregnancy and quality of life. The risk of uterine rupture, uterine incision diverticulum, and menstrual disease is significantly increased in patients with poor uterine incision healing [21]. Previous studies have suggested that uterine incision healing can be evaluated by observing the length of the uterine scar with ultrasound [19, 22]. For evaluation, each myometrial scar diameter was compared with the maximal myometrial diameter of the area previously occupied by the uterine fibroid before surgery [19]. The scar reduction index (SRI), formulated by SRI = (preoperative maximal fibroid diameter − scar length)/preoperative maximal fibroid diameter × 100%, can be used to reflect the speed of uterine incision healing after myomectomy, in which a greater SRI indicates faster uterine incision healing. This study showed that the length of the uterine scar in the PMS group (3.7 ± 1.9 cm) was smaller than that in the SCS group (5.2 ± 1.8 cm), P = 0.000, and the SRI in the PMS group (50.2%) was greater than that in the SCS group (31.0%). P = 0.000. It is suggested that seromuscular PMS can repair uterine tissue defects more accurately than SCS. However, its effect on postoperative pregnancy outcomes requires further evaluation.

In recent years, single-site laparoscopy has been extensively performed in gynecological operations, even malignancies. The most obvious advantage of single-site laparoscopy in myomectomy is the removal of fibroids through the umbilical port after bagging with avoidance of tumor dissemination. In this study, all myomectomies were performed via transumbilical single-site surgery, without any conversion to laparotomy or intraoperative complications, and no significant difference was found in the frequency of auxiliary trocars between the PMS group (5.8%) and the SCS group (3.6%). It has been shown that PMS during myomectomy can be safely and effectively completed via a transumbilical single-site laparoscopic approach. Our experience is that the surgeon’s adept suture skill is needed, especially when suturing in reverse, and certainly, it has a learning curve. Since the rotatable arm of robot-assisted laparoscopy can make suturing more convenient, robot-assisted single-site laparoscopic parallel mattress sutures during myomectomy may have potential advantages in the future [16].

Even so, considering the small sample size and the retrospective nature of this study, the results should be considered with a focus on salt, and a prospective randomized controlled trial with a large sample size is expected for further proof. Moreover, its effect on pregnancy outcomes needs further study. Whether it is appropriate for other uterine repairs, such as cesarean section and cesarean scar diverticulum, also needs further study.

## Conclusion

Parallel mattress suture of the seromuscular layer of the uterus can prevent pinhole errhysis of the uterine incision, achieve complete serosal and aesthetic incision, reduce postoperative chronic pelvic pain, and theoretically reduce the incidence of postoperative adhesions. It is safe and feasible to complete a parallel mattress suture during myomectomy via single-site laparoscopy. Further studies are required to determine pregnancy outcomes. Whether the parallel mattress suture method is suitable for other uterine repairs, such as cesarean section and uterine scar diverticulum, also needs to be further studied.

## Data Availability

All relevant data are available from the corresponding author on reasonable request.

## Abbreviations

PMS: Parallel mattress suture
SCS: Simple continuous suture
GnRHa: Gonadotropin-releasing hormone agonists
GnRHant: Gonadotropin-releasing hormone antagonists
SPRMs: Selective progesterone receptor modulators
FIGO: International Federation of Gynecology and Obstetrics
BMI: Body mass index
EBL: Estimated blood loss
SSI: Surgical site infection
SRI: Scar reduction index
ΔHb: Hemoglobin decline
ERAS: Enhanced recovery after surgery
